# Proteomic Analysis of Fractionated *Toxoplasma* Oocysts Reveals Clues to Their Environmental Resistance

**DOI:** 10.1371/journal.pone.0029955

**Published:** 2012-01-18

**Authors:** Heather M. Fritz, Paul W. Bowyer, Matthew Bogyo, Patricia A. Conrad, John C. Boothroyd

**Affiliations:** 1 Department of Pathology, Microbiology and Immunology, School of Veterinary Medicine, University of California Davis, Davis, California, United States of America; 2 Department of Microbiology and Immunology, Stanford University School of Medicine, Stanford, California, United States of America; 3 Department of Pathology, Stanford University School of Medicine, Stanford, California, United States of America; Institut National de la Santé et de la Recherche Médicale - Institut Cochin, France

## Abstract

*Toxoplasma gondii* is an obligate intracellular parasite that is unique in its ability to infect a broad range of birds and mammals, including humans, leading to an extremely high worldwide prevalence and distribution. This work focuses on the environmentally resistant oocyst, which is the product of sexual replication in felids and an important source of human infection. Due to the difficulty in producing and working with oocysts, relatively little is known about how this stage is able to resist extreme environmental stresses and how they initiate a new infection, once ingested. To fill this gap, the proteome of the wall and sporocyst/sporozoite fractions of mature, sporulated oocysts were characterized using one-dimensional gel electrophoresis followed by LC-MS/MS on trypsin-digested peptides. A combined total of 1021 non-redundant *T. gondii* proteins were identified in the sporocyst/sporozoite fraction and 226 were identified in the oocyst wall fraction. Significantly, 172 of the identified proteins have not previously been identified in *Toxoplasma* proteomic studies. Among these are several of interest for their likely role in conferring environmental resistance including a family of small, tyrosine-rich proteins present in the oocyst wall fractions and late embryogenesis abundant domain-containing (LEA) proteins in the cytosolic fractions. The latter are known from other systems to be key to enabling survival against desiccation.

## Introduction

*Toxoplasma gondii* is an important, zoonotic protozoan that can infect a wide range of warm-blooded animals, including humans. Domestic and wild felids are the only known definitive hosts in which *T. gondii* undergoes sexual replication, resulting in the formation of environmentally resistant oocysts [1], [2]. Oocysts are shed in cat feces and sporulate in the environment to become infective to other hosts, including humans. An infected cat may shed as many as one billion oocysts during a primary infection [1], [3], [4]. Oocysts are extremely durable and have been reported to survive and remain infective for years in fresh water [5] and for at least twenty-four months in salt water [6]. *T. gondii* has been emerging as a significant waterborne pathogen of public health concern, as outbreaks associated with the ingestion of contaminated water have been reported globally, including in Panama (1979), Canada (1995), French Guyana (1998), Brazil (2002) and India (2004) [7]–[11]. The association of *T. gondii* with waterborne outbreaks has led to its classification as a National Institute of Allergy and Infectious Diseases (NIAID) Category B priority agent [12].

*Toxoplasma gondii* oocysts are resistant to chemical and physical methods of inactivation used to treat waste-water and sewage [13]–[16]. Two chemicals commonly used to treat water, sodium hypochlorite (chlorine) and ozone, fail to completely inactivate infective oocysts at concentrations well in excess of those typically used to treat both sewage and drinking water [17], [18]. Although ultraviolet (UV) treatment has been shown to reduce oocyst viability, doses of 40 to 500 mJ/cm^2^ irradiation failed to inactivate all oocysts in treated water samples that were assessed by mouse bioassay [19], [20]. Even three years of storage in 2% sulfuric acid at 4°C leaves oocysts still infective to mice (data not shown). What makes the presence of these environmentally resistant oocysts an even greater health concern is that the infectious dose is very low, with experimental infections in mice and pigs resulting from exposure to as few as 1–10 oocysts [21], [22]. It is not known what structures in the oocyst wall confer the resistance to extreme environmental stresses. It is presumed that this is a result of structures present in one or both layers of the oocyst wall, but this hypothesis has not been tested.

The approximately 67 Mb genome of *Toxoplasma gondii* has been sequenced and predicts ∼8,000 genes [23], [24]. A number of proteomic studies have been conducted on both subcellular and whole *Toxoplasma* organisms, but these have all been limited to the tachyzoite life stage [25]–[27] and only about 30% of the predicted proteome has so far been detected [28], [29]. Proteomic description of the other life stages would increase the basis for validating gene predictions and provide valuable foundational and comparative data for functional analyses of identified proteins, all critical to advancing our understanding of *Toxoplasma* biology.

The aim of this study was to characterize the proteome of mature *T*oxoplasma oocyst walls and sporocysts/sporozoites, including bleach-treated versus non-bleach-treated samples to discern inner from outer wall proteins. Many novel proteins were identified in this way and the implications of our results for the environmental resistance of oocysts are discussed.

## Results

### Proteomic identification of proteins in mature oocyst fractions by LC-MS/MS

**Figure 1:**
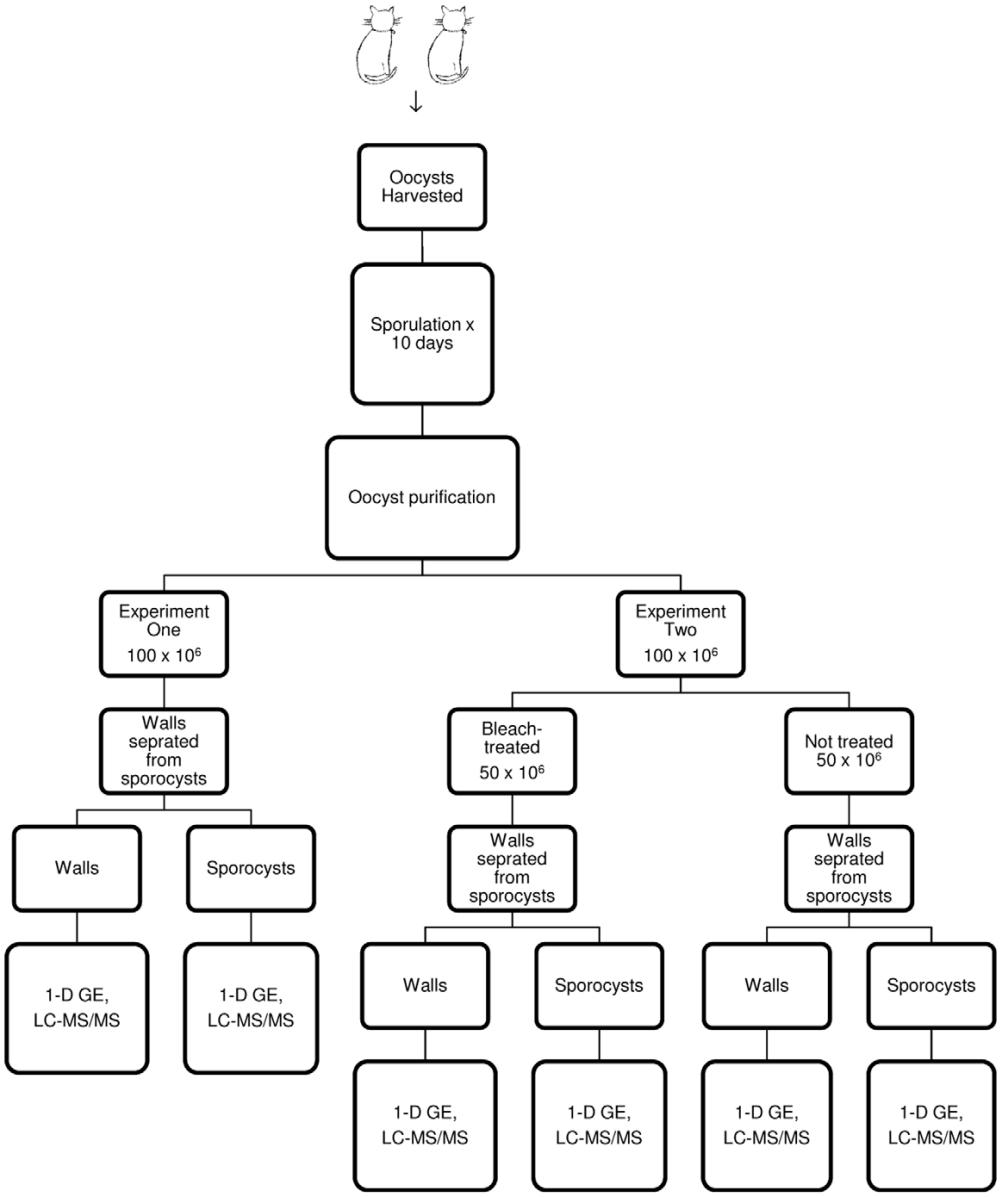
Flow chart showing experimental design for sample collection and processing of *Toxoplasma gondii* oocysts.

**Figure 2:**
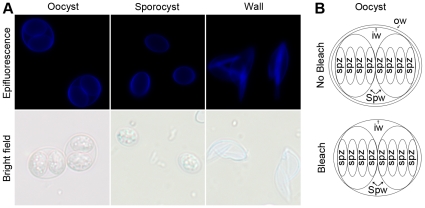
The *Toxoplasma gondii* oocyst and sporocyst walls are autofluorescent under UV excitation. A. Epifluorescent and bright field images of intact, mature oocysts 10 days after exposure to maturing conditions showing intact oocysts (“oocyst”), isolated sporocysts (“sporocyst”), and isolated oocyst walls (“wall”), the latter two fractions being derived from mature oocysts by glass bead disruption and gradient centrifugation as detailed in the materials and methods. B. A detailed schematic of the oocyst components illustrating the absence of the outer layer of the oocyst wall (ow) following treatment with bleach. The inner layer of the wall (iw) and sporocyst walls (Spw) remain intact following bleach treatment. The mature oocyst contains two sporocysts, each with 4 sporozoites (spz).

Our goal is to understand oocyst function, in particular its extreme environmental resistance, through proteomic analysis of its contents. To obtain duplicate samples for mass spectrometry, two separate experiments using 100 million oocysts each were conducted. In both experiments, oocyst walls and the sporocysts/sporozoites within were separated prior to proteomic analysis. In the second experiment, prior to wall and sporocyst/sporozoite separation, the 100 million oocysts were divided into two groups (50 million oocysts each), one that was treated with bleach to remove the outer layer of the oocyst wall and the other that was left untreated (Figures 1 and 2).

With the criterion of two-unique peptide identifications per locus, a combined total of 1031 non-redundant proteins were identified: 1021 proteins were identified in the sporocyst/sporozoite fractions and 226 in the oocyst wall fraction (Table 1, S1). Ten of the proteins identified in the wall fraction were not detected in the sporocyst/sporozoite fractions. The ToxoDB database contains a total of ∼8000 predicted proteins. We therefore detected ∼13% of all predicted proteins in the sporocyst/sporozoite fractions and ∼2% in the oocyst wall fractions. In both fractions, around 20% of the protein identifications have not previously been reported when compared with *Toxoplasma* tachyzoite MS data deposited at ToxoDB (v 6.4). All 1031 proteins identified in each experimental group, with a minimum of 2 unique peptides, are provided in Table S1. A complete list of all peptides identified in each experiment, including singlet peptides, is provided in Table S2. Proteins identified here with a minimum of two unique peptides that have not been previously identified in tachyzoites and reported on ToxoDB (v 6.4) are listed in Table S3.

**Table 1:** Summary of LC-MS/MS spectral counts and protein identification in sporocyst/sporozoite and oocyst wall fractions of *Toxoplasma gondii* oocysts.

	Experiment One	Experiment Two		
	No Bleach	Bleach	No Bleach	Totals
**Sporocysts/Sporozoites**				
Spectral counts	9613	4130	9463	23206
Protein identifications	886	438	710	1021
Decoy hits	1	0	3	4
False discovery rate	0.10%	0.00%	0.40%	0.40%
**Oocyst Walls**				
Spectral counts	2072	275	1051	3398
Protein identifications	183	53	133	226
Decoy hits	0	0	0	0
False discovery rate	0.00%	0.00%	0.00%	0.00%

### Abundantly detected proteins in sporocyst/sporozoite fractions

Proteins with the highest spectral counts in the sporocyst/sporozoite fractions, sorted on experiment one, were assembled to produce a list of the 25 most-abundantly detected proteins in sporocysts/sporozoites fractions (Table 2). Within this group of 25 proteins in the sporocyst/sporozoite fraction, there are several interesting subsets. For example, many are known to be proteins that pass through the parasite's secretory pathways leading either to the surface (glycolipid-anchored surface-antigen-one-related sequences, or SRSs) or eventually to injection into the host cell (rhoptry proteins, or ROPs and rhoptry neck proteins, or RONs) or the parasitophorous vacuole (dense granule proteins, or GRAs). Others are associated with cell recognition and adhesion (PAN-domain-containing proteins, von Willebrand factor type A domain-containing protein), movement and invasion (micronemes, actin, myosin A), carbohydrate metabolism (LDH1 isoform of lactate dehydrogenase, glyceraldehyde 3-phosphate dehydrogenase, and glucose-6-phosphate dehydrogenase) or other enzymes (oxidoreductase). Also within the list of the 25 most abundantly detected proteins are 5 hypothetical proteins. The list of all proteins confidently detected in sporocysts/sporozoites (Table S1) includes many more from these respective categories of proteins as discussed further, below.

**Table 2:** LC-MS/MS spectral counts of abundantly detected proteins in *Toxoplasma gondii* sporocyst/sporozoite fractions.

		Spectral Counts (% total spectral counts)						
		Experiment 1		Experiment 2				
		No Bleach		Bleach		No Bleach		
Gene ID^1^	Protein Description^2^	Spor^3^	Wall^3^	Spor^3^	Wall^3^	Spor^3^	Wall^3^	Prior MS^4^
058550	SRS28 (SporoSAG)	192 (2.0)	26 (1.3)	157 (3.8)	29 (10.5)	273 (2.9)	27 (2.6)	N
059670	von Willebrand factor type A domain-containing protein	164 (1.7)	6 (0.3)	94 (2.3)	12 (4.4)	241 (2.5)	32 (3.0)	N
009920	PAN domain-containing protein	135 (1.4)	12 (0.6)	94 (2.3)	19 (6.9)	184 (1.9)	34 (3.2)	N
060190	microneme protein, putative	120 (1.2)	29 (1.4)	99 (2.4)	0 (0.0)	162 (1.7)	21 (2.0)	Y
032350	lactate dehydrogenase (LDH1)	116 (1.2)	15 (0.7)	47 (1.1)	9 (3.3)	23 (0.2)	7 (0.7)	Y
112630	hypothetical protein	113 (1.2)	17 (0.8)	109 (2.6)	2 (0.7)	143 (1.5)	2 (0.2)	Y
003310	GRA7	109 (1.1)	17 (0.8)	73 (1.8)	11 (4.0)	99 (1.0)	17 (1.6)	Y
035470	myosin A, putative	106 (1.1)	19 (0.9)	51 (1.2)	10 (3.6)	115 (1.2)	23 (2.2)	Y
015780	Rhoptry kinase family protein ROP2A	106 (1.1)	7 (0.3)	57 (1.40)	0 (0.0)	88 (0.9)	12 (1.1)	Y
078830	glucose-6-phosphate dehydrogenase, putative	103 (1.1)	7 (0.3)	40 (1.0)	8 (2.9)	66 (0.7)	8 (0.8)	Y
067680	MIC12	93 (1.0)	2 (0.1)	119 (2.9)	2 (0.7)	156 (1.6)	5 (0.5)	Y
106060	RON8	88 (0.9)	5 (0.2)	18 (0.4)	4 (1.5)	58 (0.6)	6 (0.6)	Y
059900	hypothetical protein, conserved	84 (0.9)	19 (0.9)	40 (1.0)	4 (1.5)	126 (1.3)	14 (1.3)	Y
108020	SRS57	83 (0.9)	13 (0.6)	26 (0.6)	7 (2.5)	114 (1.2)	17 (1.6)	Y
094600	hypothetical protein	81 (0.80	63 (3.0)	43 (1.0)	0 (0.0)	71 (0.8)	6 (0.6)	N
023920	RON3	80 (0.8)	0 (0.0)	29 (0.7)	0 (0.0)	68 (0.7)	5 (0.5)	Y
089690	glyceraldehyde-3-phosphate dehydrogenase	75 (0.80)	18 (0.9)	24 (0.6)	2 (0.7)	30 (0.3)	6 (0.6)	Y
013280	hypothetical protein	74 (0.8)	16 (0.8)	36 (0.9)	7 (2.5)	114 (1.2)	14 (1.3)	Y
009030	actin	74 (0.8)	11 (0.5)	41 (1.0)	12 (4.4)	56 (0.6)	13 (1.2)	Y
070950	hypothetical protein	73 (0.8)	51 (2.5)	21 (0.5)	4 (1.5)	72 (0.8)	12 (1.1)	N
108080	ROP5	69 (0.7)	10 (0.5)	32 (0.8)	0 (0.0)	74 (0.8)	10 (1.0)	Y
110670	glycogen phosphorylase family protein, putative	68 (0.7)	4 (0.2)	10 (0.2)	2 (0.7)	45 (0.5)	2 (0.2)	Y
069120	oxidoreductase, putative	67 (0.7)	17 (0.8)	27 (0.7)	6 (2.2)	79 (0.8)	9 (0.9)	N
000230	PAN domain-containing protein	61 (0.6)	9 (0.4)	38 (0.9)	0 (0.0)	32 (0.3)	2 (0.2)	Y
086450	GRA5	59 (0.6)	4 (0.2)	31 (0.8)	0 (0.0)	79 (0.8)	4 (0.4)	Y

The 25 most abundant proteins in the sporocyst/sporozoite fractions sorted according to highest spectral counts in experiment 1 sporocyst/sporozoite fraction.

1 TGME49_Gene identifier according to ToxoDB.org (v6.4).

2 Identity of protein assigned by ToxoDB.org (v6.4).

3 Number of spectral counts detected for a given protein followed by the percentage that that number represents of all spectral counts in the sample indicated.

4 Previous mass spectrometry evidence of expression in tachyzoites according to ToxoDB.org (v6.4).

In a separate study (see accompanying manuscript), we performed a quantitative comparison of the transcriptomes of three major developmental stages of *Toxoplasma*: tachyzoites, bradyzoites and oocysts. In the accompanying manuscript we indicate if there was supporting proteomic evidence based on proteins detected in this study. Proteomic evidence strongly corroborates genes with high levels of mRNA detected by microarray. To facilitate the discussion of this complex dataset, we will divide the proteins into subsets based on their presumed role and/or location within the parasite.

#### Surface antigens-SRSs

The surface antigen-1-related sequences (SRSs) represent a gene family with similar structure to the major, immunodominant surface antigen, SAG1, which is abundantly expressed on tachyzoites. In the present study, 10 SRS family proteins were detected in mature oocysts (Table 3). One SRS protein was previously reported to be specifically enriched in sporozoites and was designated SporoSAG [30] or SRS28. Based on spectral counts shown in Table 2, this was the most abundantly detected protein in our analysis in each of the three tested sporozoite fractions across both experiments: 2.0% in Experiment 1 (NBT), 2.9% in Experiment 2 (NBT) and 3.8% in Experiment 2 (BT). The other SRS family genes identified in this dataset include SRS57 (SAG3), SRS51 (SRS3), SRS46, SRS29B (SAG1), SRS27B, SRS52A, SRS34A, SRS42 and a novel SRS domain-containing protein. SAG3, SRS3 and SAG1 identified in this study have previously been detected on sporozoites [30].

**Table 3:** LC-MS/MS spectral counts of functionally interesting proteins identified in sporocyst/sporozoite fractions of *Toxoplasma gondii* oocysts – SRS family proteins.

		Spectral Counts^3^			
		Experiment 1	Experiment 2		
Gene ID^1^	Protein Description^2^	No Bleach	Bleach	No Bleach	Prior MS^4^
058550	SRS28 (SporoSAG)	192	157	275	N
108020	SRS57 (SAG3)	83	26	114	Y
108840	SRS51 (SRS3)	43	10	76	Y
119350	SRS domain containing protein	31	21	44	Y
014190	SRS46	26	28	61	N
033460	SRS29B (SAG1)	17	7	18	Y
058810	SRS27B	11	5	8	N
115320	SRS52A	10	5	15	Y
071050	SRS34A (SAG2A)	7	0	9	Y
034370	SRS42	0	0	4	N

List of all SRS family proteins identified in sporocyst/sporozoite fractions.

1 TGME49_Gene identifier according to ToxoDB.org (v6.4).

2 Identity of protein assigned by ToxoDB.org (v6.4).

3 Number of spectral counts detected for a given protein.

4 Previous mass spectrometry evidence of expression in tachyzoites according to ToxoDB.org (v6.4).

#### Secretory and invasion organelles (MICs, ROPs and GRAs)

The *T. gondii* family of microneme proteins share structural domains associated with intermolecular interactions, (e.g. PAN domains, von Willebrand factor type A domain or I-domain), and are generally associated with parasite motility and invasion of host cells. Several micronemal proteins were identified in the present study, including: MIC1, MIC2, MIC2-associated protein (M2AP), MIC3, MIC4, MIC6, MIC7, MIC8, MIC10, MIC11, MIC12, MIC13, AMA1, an AMA1-paralogue (TGME49_115730), and one putative microneme protein (Table 4). Based on spectral counts, MIC12 and MIC13 were the most abundantly detected micronemal proteins in sporocyst/sporozoite fractions.

**Table 4:** LC-MS/MS spectral counts of functionally interesting proteins identified in sporocyst/sporozoite fractions of *Toxoplasma gondii* oocysts – micronemal proteins.

		Spectral Counts^3^			
		Experiment 1	Experiment 2		
Gene ID^1^	Protein Description^2^	No Bleach	Bleach	No Bleach	Prior MS^4^
060190	MIC13	120	99	162	Y
067680	MIC12	93	119	156	Y
001780	MIC2	51	29	54	Y
115730	AMA1-paralogue [SporoAMA1]	18	5	19	N
091890	MIC1	16	8	7	Y
055260	AMA1	14	5	18	Y
004530	MIC11	13	8	16	Y
014940	M2AP	10	8	7	Y
018520	MIC6	8	6	0	Y
050710	MIC10	7	4	18	Y
008030	MIC4	5	3	11	Y
045490	MIC8	4	0	3	Y
061780	MIC7	3	0	5	Y
119560	MIC3	2	0	11	Y
115550	microneme protein, putative	0	2	7	N

List of all micronemal proteins identified in sporocyst/sporozoite fractions.

1 TGME49_Gene identifier according to ToxoDB.org (v6.4).

2 Identity of protein assigned by ToxoDB.org (v6.4).

3 Number of spectral counts detected for a given protein.

4 Previous mass spectrometry evidence of expression in tachyzoites according to ToxoDB.org (v6.4).

Rhoptries are secretory organelles that are discharged during host-cell invasion. Once secreted, rhoptries may become associated with the moving junction, the parasitophorous vacuolar membrane (PVM) or the host cell nucleus where they may play a role in host cell invasion, establishment of the intracellular parasitic niche or intersecting host-cell signaling pathways, respectively [31]–[33]. In the present study, 19 rhoptry proteins were identified (Table 5). Of these, ROP2A, RON8, RON3, ROP5 and ROP42 were the five most abundant. Eight of the rhoptry proteins detected were rhoptry neck proteins (RONs), and one of these, RON2 is known to interact with AMA1 during moving junction formation and invasion by tachyzoites, [31]. Two RON2-paralogues, RON2L1 (TGME49_094400) and RON2L2 (TGME49_065120), were also detected. The three rhoptry bulb proteins, ROP2A, ROP5 and ROP42 are part of a family with a conserved kinase-fold but all three are predicted to be catalytically inactive ([34], ToxoDB.org)). Of these, ROP5 is best studied: it associates with the PVM [35] and plays a crucial but still unelucidated role in virulence [36]–[38]. ROP2A has been suggested to play a role in recruitment of host mitochondria to the PVM by tachyzoites [39] but questions about whether this is indeed its function have recently been raised [40].

**Table 5:** LC-MS/MS spectral counts of functionally interesting proteins identified in sporocyst/sporozoite fractions of *Toxoplasma gondii* oocysts – rhoptry proteins.

		Spectral Counts^3^			
		Experiment 1	Experiment 2		
Gene ID^1^	Protein Description^2^	No Bleach	Bleach	No Bleach	Prior MS^4^
015780	ROP2A	106	57	88	Y
106060	RON8	88	18	58	Y
023920	RON3	80	29	68	Y
108080	ROP5	69	32	74	Y
009980	ROP42	57	19	68	Y
005250	ROP18	44	18	39	Y
014080	Toxofilin	37	41	62	Y
100100	RON2	30	6	28	Y
010110	ROP44	17	7	34	Y
065120	RON2L2 [SporoRON2]	14	2	19	N
029010	RON4	13	9	20	Y
027810	ROP11	13	8	10	Y
058580	ROP17	13	6	15	Y
075300	ROP2B	11	5	20	Y
091960	ROP40	11	3	2	Y
111470	RON5	10	7	19	Y
115220	ROP14	9	3	9	Y
109590	ROP1	8	5	17	Y
058370	ROP28	7	2	9	N
058660	ROP6	5	0	13	Y
052360	ROP24	5	0	7	Y
010090	ROP43	4	5	5	Y
089680	Rab11	2	2	0	Y
115490	ROP10	6	0	5	Y
003990	ROP12	4	2	0	Y
097960	RON6	2	0	3	Y
094400	RON2L1	6	0	2	N
011290	ROP15	5	0	0	Y
040090	ROP34	0	0	5	Y
058800	ROP31	0	0	2	Y

List of all rhoptry proteins identified in sporocyst/sporozoite fractions.

1 TGME49_Gene identifier according to ToxoDB.org (v6.4).

2 Identity of protein assigned by ToxoDB.org (v6.4).

3 Number of spectral counts detected for a given protein.

4 Previous mass spectrometry evidence of expression in tachyzoites according to ToxoDB.org (v6.4).

Dense granule proteins are discharged toward the end of the invasion process and remain freely soluble or associate either with the parasitophorous vacuolar membrane (PVM) or the membranous intravacuolar network (IVN), which is linked to the PVM [41]. They represent a diverse family of proteins with little stage-specificity [42], [43]. Their function is generally not well understood but probably has to do with maintenance of the PVM. In vitro studies indicate that upon entry into the host cell, sporozoites form a temporary parasitophorous vacuole (PV1) before moving into a second parasitophorous vacuole (PV2) where the majority of dense granule proteins are secreted. GRA3 and GRA5 were previously found to be secreted into PV1 and PV2, while NTPase, GRA1, GRA2, GRA4, and GRA6 were exclusively secreted into PV2 [44]. All are abundantly present in the tachyzoite PV [44]. Eight dense granule proteins were identified in the present dataset, including GRA1, GRA2, GRA4, GRA5, GRA6, GRA7, GRA8 and GRA14 (Table 6).

**Table 6:** LC-MS/MS spectral counts of functionally interesting proteins identified in sporocyst/sporozoite fractions of *Toxoplasma gondii* oocysts – dense granule proteins.

		Spectral Counts^3^			
		Experiment 1	Experiment 2		
Gene ID^1^	Protein Description^2^	No Bleach	Bleach	No Bleach	Prior MS^4^
003310	GRA7	109	73	99	Y
086450	GRA5	59	31	79	Y
039740	GRA14	17	7	23	Y
110780	GRA4	16	11	17	Y
075440	GRA6	9	7	20	Y
054720	GRA8	8	0	2	Y
070250	GRA1	4	0	9	Y
027620	GRA2	2	0	2	Y

List of all dense granule proteins identified in sporocyst/sporozoite fractions.

1 TGME49_Gene identifier according to ToxoDB.org (v6.4).

2 Identity of protein assigned by ToxoDB.org (v6.4).

3 Number of spectral counts detected for a given protein.

4 Previous mass spectrometry evidence of expression in tachyzoites according to ToxoDB.org (v6.4).

#### Other proteins of interest

Additional proteins considered to be of interest are listed in Table 7. Among these are four “late-embryogenesis abundant domain-containing” (LEA) proteins that have not previously been detected in proteomic studies of bradyzoites and tachyzoites (as reported on ToxoDB v6.4). Two metabolic enzymes (lactate dehydrogenase (LDH1) and enolase (ENO2)) and a superoxide dismutase (SOD3) were also detected. The two isoenzymes associated with carbohydrate metabolism, LDH1 and ENO2, were previously reported to be abundantly expressed in *Toxoplasma* tachyzoites but not bradyzoites [42], [45], [46].

**Table 7:** LC-MS/MS spectral counts of functionally interesting proteins identified in sporocyst/sporozoite fractions of *Toxoplasma gondii* oocysts – other proteins of interest.

		Spectral Counts^3^			
		Experiment 1	Experiment 2		
Gene ID^1^	Protein Description^2^	No Bleach	Bleach	No Bleach	Prior MS^4^
032350	LDH1	116	47	23	Y
068850	ENO2	38	31	30	Y
116190	Superoxide dismutase, putative (SOD3)	23	10	11	N
076860	late embryogenesis abundant domain-containing protein	24	18	43	N
076850	late embryogenesis abundant domain-containing protein (TgERP)	20	39	56	N
076880	late embryogenesis abundant domain-containing protein	12	27	36	N
076870	LEA1 protein, putative	34	7	34	N

List of other proteins identified in sporocyst/sporozoite fractions that were considered to be of interest.

1 TGME49_Gene identifier according to ToxoDB.org (v6.4).

2 Identity of protein assigned by ToxoDB.org (v6.4).

3 Number of spectral counts detected for a given protein.

4 Previous mass spectrometry evidence of expression in tachyzoites according to ToxoDB.org (v6.4).

### Abundantly detected proteins in oocyst wall fractions

Fractionation allows information to be gleaned about a protein's likely location and, therefore, clues to its possible function. In parallel to the analysis of the sporocyst/sporozoite fractions above, we also analyzed fractions enriched for oocyst walls. The proteins with the greatest number of spectral counts in these latter fractions were two PAN-domain-containing proteins and a putative oxidoreductase (Table 8). Also high on the list of abundant proteins in the wall-enriched fractions were a putative micronemal protein with PAN-domains (TGME49_054430), another PAN-domain-containing protein, four tyrosine-rich hypothetical proteins, a putative GPI transamidase subunit (PIG-U), a putative oocyst wall protein (based on homology to a *Cryptosporidium* oocyst wall protein), and a putative alanine dehydrogenase.

**Table 8:** LC-MS/MS spectral counts of most abundantly detected proteins in *Toxoplasma gondii* oocyst wall fractions showing spectral counts (% of all spectral counts) in each experimental group.

		Spectral Counts (% total spectral counts)									
		Experiment 1			Experiment 2						
		No Bleach			Bleach			No Bleach			
Gene ID^1^	Protein Description^2^	Wall^3^	Spor^3^	Fold^4^	Wall^3^	Spor^3^	Fold^4^	Wall^3^	Spor^3^	Fold^4^	Prior MS^5^
035200	PAN domain-containing protein	391 (18.9)	18 (0.2)	100.8	0 (0.0)	0 (0.0)	N/A^6^	150 (14.3)	23 (0.2)	58.7	N
053150	oxidoreductase, putative	217 (10.5)	13 (0.1)	77.4	0 (0.0)	0 (0.0)	N/A	147 (14.0)	45 (0.5)	29.4	N
035390	PAN domain-containing protein	144 (6.9)	0 (0.0)	N/C^7^	0 (0.0)	0 (0.0)	N/A	41 (3.9)	3 (0.0)	123.1	N
054430	microneme protein, putative	75 (3.6)	0 (0.0)	N/C	0 (0.0)	0 (0.0)	N/A	3 (0.3)	0 (0.0)	N/C	N
009470	hypothetical protein	36 (1.7)	35 (0.4)	4.8	5 (1.8)	13 (0.3)	5.8	17 (1.6)	65 (0.7)	2.4	N
035210	PAN domain-containing protein	32 (1.5)	0 (0.0)	N/C	0 (0.0)	0 (0.0)	N/A	5 (0.5)	0 (0.0)	N/C	N
051910	hypothetical protein	29 (1.4)	0 (0.0)	N/C	0 (0.0)	0 (0.0)	N/A	2 (0.2)	4 (0.0)	4.5	N
077570	GPI transamidase subunit PIG-U, putative	29 (1.4)	12 (0.1)	11.2	0 (0.0)	9 (0.2)	0.0	5 (0.5)	41 (0.4)	1.1	Y
037080	hypothetical protein*	27 (1.3)	3 (0.0)	41.8	2 (0.7)	5 (0.1)	6.0	2 (0.2)	4 (0.0)	4.5	N
094820	type I fatty acid synthase, putative	18 (0.9)	12 (0.1)	7.0	0 (0.0)	0 (0.0)	N/A	0 (0.0)	0 (0.0)	N/A	Y
087250	hypothetical protein*	13 (0.6)	3 (0.0)	20.1	0 (0.0)	3 (0.1)	0.0	3 (0.3)	3 (0.0)	9.0	N
081590	hypothetical protein*	12 (0.6)	6 (0.1)	9.3	0 (0.0)	13 (0.3)	0.0	10 (1.0)	20 (0.2)	4.5	N
003500	alanine dehydrogenase putative	8 (0.4)	3 (0.0)	12.4	0 (0.0)	0 (0.0)	N/A	0 (0.0)	0 (0.0)	N/A	N
069380	hypothetical protein	6 (0.3)	5 (0.1)	5.6	0 (0.0)	2 (0.0)	0.0	2 (0.2)	6 (0.1)	3.0	N
106050	hypothetical protein	5 (0.2)	4 (0.0)	5.8	3 (1.1)	4 (0.1)	11.3	4 (0.4)	0 (0.0)	N/C	N
048810	hypothetical protein	5 (0.2)	5 (0.1)	4.6	0 (0.0)	2 (0.0)	0.0	3 (0.3)	2 (0.0)	13.5	Y
058910	hypothetical protein	5 (0.2)	0 (0.0)	N/C	0 (0.0)	0 (0.0)	N/A	0 (0.0)	0 (0.0)	N/A	N
032170	hypothetical protein	5 (0.2)	0 (0.0)	N/C	0 (0.0)	0 (0.0)	N/A	0 (0.0)	7 (0.1)	0	Y

Abundant proteins in the oocyst wall fractions sorted first according to highest spectral counts in experiment 1 walls, then on fold-enrichment in wall fraction. Criteria for inclusion in table are a minimum of 5 spectral counts in at least one of the wall fractions and a minimum of 5-fold-enrichment in one or both no bleach wall samples.

1 TGME49_Gene identifier according to ToxoDB.org (v6.4).

2 Identity of protein assigned by ToxoDB.org (v6.4).

3 Number of spectral counts detected for a given protein in wall or sporocyst/sporozoite fraction.

4 Fold-enrichment in the wall fraction. Calculation: [(spectral counts for a given protein in wall fraction)/(total spectral counts in wall fraction)] divided by [(spectral counts for a given protein in sporocyst/sporozoite fraction)/(total spectral counts in sporocyst/sporozoite fraction)].

5 Previous mass spectrometry evidence of expression in tachyzoites according to ToxoDB.org (v6.4).

6 N/A is “not applicable” and is designated when no fold enrichment exists because no spectral counts are detected in both wall and sporocyst/sporozoite fractions.

7 N/C is “not calculable” and is designated when a zero is present in the denominator of the equation. Where N/C is reported spectral counts were detected in the wall fraction and not the sporocyt/sporozoite fraction.

*Tyrosine-rich protein (>5% tyrosine).

It is expected that wall components will also be detected within sporocyst/sporozoite fractions because their deposition in the wall could be a continuous process and so new material destined for this structure could be caught “in transit”. As well, whenever preparing subcellular fractions, a certain amount of contamination in the separated fractions is inevitable. A very large ratio of spectral counts in the oocyst wall relative to the sporocyst/sporozoite fractions, however, should be indicative of a true localization in the wall. Ten proteins were identified exclusively in the wall fractions. To interrogate the specific enrichment of abundant proteins detected in oocyst wall fractions, compared to the sporocyst/sporozoite fraction within each experimental group, a fold-enrichment calculation was made using the following formula: [(spectral counts for a given protein in wall fraction)/(total spectral counts in wall fraction)] divided by [(spectral counts for a given protein in sporocyst/sporozoite fraction)/(total spectral counts in sporocyst/sporozoite fraction)]. The protein identifications included in table 8 represented those with the most abundant spectral counts identified in oocyst wall fractions and evidence of enrichment in the walls (greater than 5-fold enrichment in walls in at least one of the two “no bleach” samples, compared to corresponding sporocyst/sporozoite fraction). Although not strictly quantitative, the spectral counts mapping to a given protein relate to the abundance of that protein and comparisons for an individual protein, when made between the oocyst wall fractions and sporocyst/sporozoite fractions, should not be substantially affected by that protein's size or ease of detection by MS [47]. Clearly, other factors can impact the detectability of a protein, such as the abundance and nature of other proteins in the fraction, but this approach provides at least an initial estimate of the relative enrichment of a protein in the oocyst wall fractions.

Bleach-treatment has been reported to strip oocysts of the outer-most layer of the wall [48] and so this treatment was applied to half the starting material in experiment two; proteins present in the outer layer of the oocyst wall should therefore be present in the untreated samples but depleted in the bleach-treated material. We found that following treatment with bleach, the total number of spectral counts and protein identifications obtained for the wall fraction dropped by 73.8% and 60.2%, respectively. Importantly, however, bleach treatment appeared to reduce the sensitivity of the assay overall as the spectral counts and protein identifications in sporocyst/sporozoite fractions were also reduced following bleach treatment by 56.4% and 38.3%, respectively. As a result, conclusions about whether a given protein in the oocyst wall is likely from the inner versus outer layers are tentative and limited to those proteins where the differential was extreme; i.e., proteins with very high spectral counts in both non-bleach-treated fractions and zero spectral counts in the corresponding bleach-treated fractions. Based on this criterion, the top three proteins listed in Table 8 (two PAN domain-containing proteins, and a putative oxidoreductase) appear most likely to be located in the outer layer of the wall.

### Tyrosine-rich, “Eimeria gam-like” proteins and oocyst wall proteins

Prior to this work, the proposed model for oocyst wall composition and formation in *Toxoplasma* was primarily derived from studies describing the corresponding structure in the closely related coccidian *Eimeria*. The *Eimeria* model relies upon the presence of tyrosine-rich proteins that are believed to be cross-linked via tyrosine residues through peroxidase activity [49]. A BLAST search of the predicted *Toxoplasma* proteome (ToxoDB.org) with sequence similarity to two *Eimeria* wall proteins, EmGam56 and EmGam82, failed to identify any compelling homologues (data not shown). A search of the predicted *Toxoplasma* proteome for tyrosine-rich proteins with a signal sequence, however, did identify several hypothetical proteins. This was done by searching all predicted proteins in the *Toxoplasma* proteome found on ToxoDB (v6.4) for their amino acid content using tools available at the following website: http://pir.georgetown.edu/pirwww/search/comp_mw.shtml. The percent tyrosine cut-off was set at 5% with the aim to capture those proteins near and above the EmGam56 and 82 percentages, which are 8.6% and 9.7% tyrosine, respectively (determined from published translation sequences: AAN05087.1 and AAO47083.2) [50], [51]. Several proteins with >5% tyrosine were identified by this search method. Presented here are six that were both identified in the oocyst proteome and also had supporting evidence of expression in the oocyst stage by microarray (see accompanying manuscript). These proteins were detected in the oocyst wall fractions analyzed in this study (Table 9); however, they were also detected in the sporocyst/sporozoite fractions. None of them have been detected in previous reports of tachyzoite proteomes (ToxoDB v6.4). Because they were detected in both sporocyst/sporozoite and oocyst wall fractions it is not clear if these are oocyst wall components and/or sporocyst wall components, or serve some other function.

**Table 9:** LC-MS/MS spectral counts of tyrosine-rich (>5%) and putative oocyst wall proteins identified in sporocyst/sporozoite and wall fractions of *Toxoplasma gondii* oocysts.

		Experiment 1	Experiment 2					
		No Bleach		Bleach		No Bleach		
Gene ID^1^	Protein Description^2^	Wall^3^	Spor^3^	Wall^3^	Spor^3^	Wall^3^	Spor^3^	Prior MS^4^
**Tyrosine-rich proteins (% tyrosine)**								
116550	hypothetical protein (19.5%)	2	6	0	2	0	7	N
081590	hypothetical protein (15.5%)	12	6	0	13	10	31	N
087250	hypothetical protein (13.5%)	13	3	0	3	3	6	N
037080	hypothetical protein (6.2%)	27	3	2	5	2	9	N
120530	hypothetical protein (5.6%)	0	2	0	0	0	3	N
119890	hypothetical protein (5.5%)	9	50	10	70	22	47	N
**Putative oocyst wall proteins**								
086250 (TgOWP6)^5^	oocyst wall protein, putative	30	36	0	18	12	44	N
009610 (TgOWP2)^5^	oocyst wall protein COWP, putative	18	29	0	17	2	38	N

List of tyrosine-rich and putative oocyst wall proteins identified in wall and sporocyst/sporozoite fractions.

1 TGME49_Gene identifier according to ToxoDB.org (v6.4).

2 Identity of protein assigned by ToxoDB.org (v6.4).

3 Number of spectral counts detected for a given protein in the wall and sporocyst/sporozoite fraction.

4 Previous mass spectrometry evidence of expression in tachyzoites according to ToxoDB.org (v6.4).

5 Designations for COWP homologues in *Toxoplasma* by Possenti et al. [59].

## Discussion

We report here the first comprehensive inventory of oocyst proteins in *Toxoplasma*, with preliminary indications of which proteins are in the oocyst wall versus the sporocyst/sporozoite themselves and which of the wall proteins are inner versus outer wall components. While a definitive conclusion about the location of any one protein cannot be made from these data, there are many trends that are unambiguous. Many proteins that have been previously well studied in terms of location and function in tachyzoites and bradyzoites were abundantly detected in our oocyst data set. All of the previously described “tachyzoite-specific” SRS proteins (i.e., abundantly expressed in tachyzoites but not bradyzoites), but none of the “bradyzoite-specific” SRS proteins were identified in our data set. These findings are in agreement with prior studies identifying SAG1, SAG3 and SRS3 on the surface of sporozoites [30] and strongly reinforce the transcriptomic data in the accompanying manuscript that showed sporozoites have more of a tachyzoite than bradyzoite phenotype in terms of SRS gene expression.

The data reported here suggest that, as with the surface antigens, the metabolic proteins of sporozoites may be more similar to tachyzoites than bradyzoites. The ENO2 isoform of enolase and LDH1 isoform of lactate dehydrogenase, which are known to be much more abundantly expressed in tachyzoites relative to bradyzoites, were detected in this oocyst study whereas ENO1 and LDH2 that predominate in bradyzoites were not detected [42], [45], [46]. This is not surprising given that immunolocalization studies of the ENO and LDH isoforms in bradyzoites and tachyzoites and coccidian stages within the feline intestine showed that, like tachyzoites, coccidian stages stain positive for ENO2 and LDH1 and negative for the bradyzoite-abundant ENO1 and LDH2 [46]. This may be an indication that the metabolism of freshly sporulated sporozoites is both retained from the intestinal sexual stages and is more similar to the rapidly dividing tachyzoite than to the generally more quiescent bradyzoite. Along similar lines, there appears to be a superoxide dismutase isoform that is specific to the oocyst, SOD3 [52]. The SOD3 isoform has not been detected in proteomic analyses of tachyzoites (ToxoDB v6.4), suggesting a function unique to the oocyst, perhaps protection against oxidative stresses experienced in the environment.

The most abundantly detected microneme protein in our dataset was MIC13. MIC13 is a recently described microneme protein in *T. gondii*. It has three microneme adhesive repeat (MAR) domains known to bind sialylated glycoconjugates on host cells [53]. Conservation of the MAR domain among coccidian enteroparasites suggests a specific role in enteric invasion; as such it has been proposed that binding of sialylated glycoconjugates on host cells facilitates invasion through the gut epithelium [53]. Another microneme protein possessing MAR domains is MIC1 [54]. It was shown in tachyzoites that MIC1 operates in a complex with two other micronemal proteins, MIC4 and MIC6, to create the TgMIC1-4-6 complex [55]. Each micronemal protein in the MIC1-4-6 complex possesses unique domains, including MAR domains in MIC1, Epidermal Growth Factor-like domains (EGF1) in MIC6, and PAN-apple domains in MIC4 [55], [56]. MIC13 was shown to traffic to the micronemes independent of the MIC1-4-6 complex and it was proposed that it belongs to its own complex of micronemal proteins [53]. Given the separate but synergistic roles of the proteins in the MIC1-4-6 complex, it might logically follow that MIC13 would similarly form complexes with other micronemal proteins. Other micronemal proteins abundantly detected in the sporocyst/sporozoite fractions that could ostensibly participate in such a complex include: two microneme proteins containing EGF domains, MIC12 and an additional, putative microneme protein (TGME49_115550); a putative microneme protein with PAN domains (TGME49_060190); and two PAN-domain-containing proteins, (TGME49_009920 and TGME49_000230), which have not been localized to the micronemes, requiring further investigation into their roles and locations.

Transcriptomic analyses indicated that paralogues of two proteins that are key to moving junction formation, AMA1 and RON2, are expressed in sporozoites, at least at the RNA level (see accompanying manuscript). These paralogues, dubbed SporoAMA1 and SporoRON2, were readily detected in the oocyst proteome reported here but so too were the original AMA1 and RON2 proteins, as well as all the other members of the moving junction complex, RON4, RON5 and RON8. This suggests that sporozoites might have two options for forming a moving junction during host-cell invasion. Dissecting the true functions of this putative, alternative pairing will require extensive further work. PAN domain-containing proteins are known to play a role in protein-protein and protein-carbohydrate interactions [57]. The structural conformation of PAN-domain containing proteins is achieved through disulfide bridges resulting in a pattern of folding that creates recognition and binding sites [58]. Two PAN domain-containing proteins were uniquely abundant in the oocyst wall by MS (TGME49_035200 and TGME49_035390) and two were enriched in the sporocyst/sporozoite fractions (TGME49_009920 and TGME49_000230), the significance of which is not clear. Current thinking is that sporozoites excyst from oocysts in the gastric environment, followed by invasion into host intestinal cells but these processes have not been studied in any detail *in vivo*. Therefore, the possibility that the oocyst engages in host cell recognition and enterocyte attachment prior to release of sporozoites should not be excluded. In addition, it is possible that the PAN domain-containing proteins in the wall are of structural significance given their large size and predicted disulfide bridges. As discussed above, a micronemal location for the PAN domain-containing proteins in the sporozoite seems likely but has yet to be demonstrated.

Studies of oocyst wall composition in the closely related coccidian *Eimeria* and the gregarine-like *Cryptosporidium* have been previously performed. Both an *Eimeria*-like oocyst wall, composed of tyrosine-rich proteins held together by tyrosine cross-links, and a *Cryptosporidium*-like oocyst wall, composed of cysteine-rich proteins held together by disulfide bonds, are supported by our data. Interestingly, the two OWPs (TgOWP2 and TgOWP6) identified in the present study were detected in both the oocyst wall and sporocyst/sporozoite fractions. The presence of OWPs in the sporocyst/sporozoite and wall proteomes reported here could reflect proteins in transit to the wall, although as the oocyst wall appears to be fully formed in even immature oocysts, it seems unlikely that they would still be being synthesized in mature oocysts if this is their only purpose. Instead, it could be that these proteins are part of the sporocyst wall, which is not present in immature oocysts, or they could be exclusively oocyst wall proteins and simply be contaminants of the sporocyst/sporozoite fractions due, for example, to their large size, likely affinity with other proteins and/or relative abundance. In support of the latter explanation, TgOWPs 1, 2 and 3 were all previously identified as oocyst wall, but not sporocyst wall, components [59], suggesting that the TgOWP2 detected in the sporocyst/sporozoite fractions was a contaminant from the wall fraction.

The finding of several tyrosine-rich proteins offers a possible explanation for the observed autofluorescence of *T*oxoplasma oocyst walls. The data here do not allow us to address whether these proteins are cross-linked through their tyrosines (a linkage known to produce autofluorescence) but their abundance and location makes this a distinct possibility. Furthermore, the peroxidase homologues detected in the oocyst wall fraction (TGME49_053150) and the sporocyst/sporozoite fraction (TGME49_069120) could provide the catalytic machinery involved in the cross-linking. Resolution of both possibilities will await more detailed structural and biochemical studies.

Sporocyst walls might be predicted to have similar proteins because they are also autofluorescent. The tyrosine-rich proteins described here were detected in both the wall and sporocyst/sporozoite fractions. Enrichment calculations (Table 8) suggest that at least three of the tyrosine-rich proteins are enriched in the oocyst wall fraction. We could not determine if the tyrosine-rich proteins in the sporocyst/sporozoite fraction were a contaminant or if they were in the sporocyst walls or sporozoites within. Interestingly, the genes encoding the two tyrosine-rich proteins most enriched in the oocyst wall were also most highly expressed in d0 oocysts relative to d4 and d10, suggesting that these proteins may be present at the time of wall formation, preceding formation of sporocyst walls. Similarly, the genes encoding the two tyrosine-rich proteins that appeared to be most enriched in sporocyst/sporozoite fractions were most highly expressed in d4 oocysts, at the time sporocysts are being formed. If this latter set of proteins is in the sporocyst wall and if they do in fact play a role in structural robustness and resistance to inactivation, it might be that these structures provide an additional level of resistance to chemical and physical inactivation.

A particularly intriguing group of proteins, designated “late-embryogenesis abundant domain-containing” proteins (LEAs), were identified here. While the function of these proteins in *T. gondii* is unknown, LEA proteins have been described in a number of other organisms including plants, invertebrates and microorganisms [60]. There is significant diversity in the LEA families and their respective functions are still under investigation. However, a commonly ascribed role is in resistance to environmental stresses including drought, high salinity and freezing [61]. One of the LEA proteins in *T. gondii* was recently identified as a sporozoite-specific antigen and named “*Toxoplasma gondii* embryogenesis-related protein” (TgERP) and corresponds to the gene TGME49_076850 [62]. While it has been presumed that the *T. gondii* oocyst achieves its resistance to environmental destruction through structures present in the wall, the finding that the LEA proteins are both abundant in oocysts and at least one is immunogenic to the host implies that LEA proteins may be another critical component to the oocyst that should receive further attention.

Further studies examining the oocyst through its development will be useful to determine if oocyst wall composition changes as the oocyst matures and sporulates once outside of the feline definitive host and in the environment. There is reason to believe that it might: the oocyst goes from very weakly autofluorescent when first shed in feces as the unsporulated stage to intensely autofluorescent at maturity as a sporulated oocyst (data not shown) and the oocyst becomes more resistant to disinfectants as it matures [63]. Whether additional proteins are incorporated as the oocyst matures or if existing proteins are simply modified (e.g. tyrosine cross-linked) is not known.

Functional analysis of newly identified proteins, such as the LEAs, is also critical to advancing our understanding of the oocyst as an environmentally resistant stage. The knowledge gained from these studies will be useful to develop antibody-based methods for oocyst concentration and detection in water, like those used in Environmental Protection Agency-approved methods for *Giardia* and *Cryptosporidium*, as well as strategies for more effective oocyst inactivation that could be applied to water treatment. The inventory of oocyst wall and internal oocyst proteins reported here, 172 of which have not been previously detected in the tachyzoite proteomes (Table S3), represents a crucial first step to complete dissection of this under-studied but highly important stage in the biology of *Toxoplasma gondii*.

## Materials and Methods

### Ethics statement

All kitten and mouse experiments were conducted conforming to the guidelines of the Animal Welfare Act and the Health and Research Extension Act. All experimental protocols specific to this study were approved by the UC Davis Institutional Animal Care and Use Committee (IACUC approval #15619), which is accredited by the Association for Assessment and Accreditation of Laboratory Animal Care International. Efforts were made to minimize the numbers of animals used to generate *Toxoplasma* organisms. The kittens used in the study remained healthy throughout. After two weeks of confirmed absence of shedding of *Toxoplasma* oocysts, the kittens were vaccinated and neutered, then adopted out to pre-screened and approved permanent homes.

### Parasite Strain and Infection of Mice

As described in the accompanying manuscript (Fritz et al., submitted), for all of the studies described here, we used the M4 strain of *T. gondii*, originally isolated from an aborted sheep fetus and kindly provided to our laboratory by Lee Innes of the Moredun Research Institute, Edinburgh, Scotland. This isolate was genetically characterized as a type II isolate (see accompanying manuscript). To obtain oocysts for these experiments kittens were infected by feeding infected mouse brains containing bradyzoite cysts, as described in detail in accompanying manuscript.

### Oocyst Harvest from Kitten Feces and Sporulation

Kittens were screened for oocyst shedding and oocysts were harvested from infected kitten feces using methods identical to those described in the accompanying manuscript [64]. Briefly, feces were examined for oocyst shedding by zinc sulfate double centrifugation [3] and oocysts were harvested from feces by sodium chloride flotation [17]. Concentrated oocyst pellets were resuspended in approximately 12 ml of 2% sulfuric acid and transferred to a T75 tissue culture flask for sporulation by aeration and gentle shaking at room temperature (∼22°C) for 10 days.

### Oocyst Purification

Sporulated oocysts were collected from T75 sporulation flasks, washed with PBS to restore neutral pH and purified using gradient separation of oocysts with cesium chloride (CsCl) in Tris-EDTA (TE) buffer (10 mM Tris-Cl, 1 mM EDTA, pH 7.4), as described in accompanying manuscript. Purified oocysts were enumerated using a hemocytometer and a total of 100 million oocysts were used for each experiment.

### Treatment with bleach to remove the outer-most layer of the oocyst walls

In experiment two, 100 million oocysts were split into two aliquots: bleach-treated (BT) and non-bleach treated (NBT). Bleach-treated oocysts were suspended in 50% Clorox® bleach (1∶1 PBS and Clorox) and gently agitated at room temperature for 30 minutes. Clorox® contains 5–10% sodium hypochlorite. The oocysts were then washed three times with PBS to remove bleach.

### Oocyst Wall and Sporocyst Separation

CsCl-purified oocysts were disrupted using acid-washed glass beads (350 mg, 200–400 mm, Invitrogen) in 1.5 ml screw-top microcentrifuge tubes and vortexed at max speed in 20 sec intervals until >90% of oocysts were broken open with visible walls (roughly 60–120 sec total vortex time). Walls and sporocysts/sporoblasts were purified using OptiPrep (SIGMA) reagents for gradient separation, according to methods similar to those previously described [65], [66]. Oocyst walls were harvested at the 25%/30% interface and sporocysts were harvested at the 5%/15% interface. Walls and sporocysts were washed three times in PBS, with the first two spins being done at 2500×g for 15 min and the pellets consolidated at each step. The pellet from the second wash was transferred to a 1.5 ml microcentrifuge tube and a final wash was done, spinning at 14,000 rpm for 8 min. Samples were resuspended in 500 µl PBS and stored immediately at −80°C until processed for mass spectrometry.

### Protein Preparation and Mass Spectrometry (MS)

In order to safely work with oocyst preparations in the MS laboratory, it was necessary to inactivate oocyst materials and demonstrate their non-infectivity. To do this, oocyst fraction preparations were subjected to three freeze-thaw cycles, (2 minutes each: 100% ethanol-dry ice bath followed by room temperature water bath). Evaluation of this method in our laboratory confirmed that oocysts were effectively inactivated as determined by mouse bioassay.

### 1-D SDS-PAGE

Oocyst fractions, (100×10^6^ oocyst equivalents in experiment one and 50×10^6^ in each bleach-treated or not bleach-treated sample in experiment two), were concentrated by filtration through a 3 kDa filter, resuspended in 1× LDS lysis buffer (lithium dodecyl sulfate, pH 8.4, Invitrogen) heated to 96°C for 5 min, and then separated on a 7 cm long, 4–12% NuPAGE Bis-Tris gel (Invitrogen) in 1× MES (2-(*N*-morpholino)ethanesulfonic acid: 50 mM MES, 50 mM Tris Base, 0.1% SDS, 1 mM EDTA, pH 7.3, Invitrogen) running buffer. The gel was run at a constant voltage (150 V) for approximately one hour. The entire length of each sample lane was cut into 14, 0.5 cm, slices. Gel slices were subjected to in-gel digestion with trypsin as described [67] before analysis on an LTQ ion trap mass spectrometer (ThermoFisher).

### LC-MS/MS

LC-MS/MS and subsequent analysis was performed as described [68]. Briefly, peptides were separated on a Basic Picofrit C_18_ capillary column coupled to an Eksigent nanoLC-2D™ pump before analysis on an LTQ ion trap mass spectrometer (ThermoFisher). Peptides were eluted with an acetonitrile gradient from 0 to 60% in a 0.1% solution of formic acid over 2 hr. The flow rate through the column was 250 nl/min and the spray voltage was 2.0 kV. Data-dependent scanning was employed allowing six MS2 scans of the most abundant ions of the parent full MS scan. Dynamic exclusion was enabled for 180 sec.

### Data Analysis

RAW files were generated for each gel slice by XCalibur (ver 2.0) running in conjunction with the mass spectrometer. These were analyzed using the Sequest algorithm in Bioworks (ver 3.3) software package. Searches were performed against a custom concatenated target-decoy database containing the annotated proteins based on the *T. gondii* ME49 release 5 sequences (http://toxodb.org) and *Cryptosporidium parvum* database (http://cryptodb.org). The *C. parvum* database was searched because prior to infection with *Toxoplasma*, the kittens used in this experiment were found to be shedding small numbers of *Cryptosporidium* oocysts in their feces, as detected in the pre-infection screening of fecal flotations. No *Cryptosporidium* oocysts were observed once *Toxoplasma* oocysts were detected and subsequently harvested and purified. Very few *C. parvum* protein matches were detected in the database search and those were exclusively in abundant, highly conserved proteins (e.g., ribosomal and heat shock proteins – see tables S1 and S2). Given that they were from such highly conserved proteins, their exact biological origin could not be known with certainty. Based on exclusion criteria in the analysis, all identifications with a match in more than one database (CryptoDB and ToxoDB) were removed from the analysis. The few *Cryptosporidium* sequences that were identified were computationally excluded from further analysis reported herein. It is possible that other highly conserved proteins were identified in this dataset and designated as either *Cryptosporidium* or *Toxoplasma* but may have actually been peptides of another origin (ie. proteins from the cat present in the samples) but were not excluded because that database was not searched. Negative exclusion criteria could not be run for the cat genome because it is not available. As a result of the removal of highly conserved proteins in *Toxoplasma* and *Cryptosporidium*, they will be under-represented in these data. As the highly conserved proteins were not the focus of interest in this study their exclusion are not expected to impact the conclusions. SEQUEST data from each gel band were filtered and sorted using DTASelect version 1.9 [69] under default settings. Peptides in the +1, +2 and +3 charge-states were required to have minimum XCorr values of 1.8, 2.5 and 3.5, respectively. The minimum requirement for deltaCN was 0.08. Table S2 shows all peptides identified. Both tryptic and semi-tryptic peptides are shown. The false discovery rate (FDR) was determined according to the guidelines of Elias and Gygi [70]. For the complete combined peptide data, not including singlet peptides, the FDR was 0.19%. For the complete protein identification (Table S1), the FDR was 0.85%. The FDR for all peptides detected, with singlet peptides included, was 14%. In all discussion within the manuscript, only the data with a requirement of two unique peptide identifications per protein were considered.

### Data Deposition

The data associated with this manuscript may be downloaded from the ProteomeCommons.org Tranche network using the following hash: Qw91UWAR++RGze0MZALyCRMob/x1n3Ng+KsCQ284fZq2VnI9gr/Z0cnCfjAVo7KDt3503RYwfR2LYo0w/WeVi6VHOJsAAAAAAABX7A =  = . The peptide identifications have also been made publicly available on ToxoDB (www.ToxoDB.org).

## Supporting Information

Table S1 **All proteins identified by LC-MS/MS with a minimum of two unique peptides.**
(XLSX)

Table S2 **All tryptic and semi-tryptic peptides identified by LC-MS/MS, singlet peptides included.**
(XLSX)

Table S3 **All proteins identified for which no prior MS evidence has been detected and reported on ToxoDB.org (v6.4).**
(XLSX)
